# Voltammetric Investigation of Ferulic Acid at Disposable Pencil Graphite Electrode

**DOI:** 10.3390/mi14101951

**Published:** 2023-10-19

**Authors:** Iulia Gabriela David, Dana Elena Popa, Mihaela Buleandra, Silvia Nicoleta Codreanu, Lorelei Croitoru, Laura Andreea Iordache, Hassan Noor

**Affiliations:** 1 Department of Analytical Chemistry and Physical Chemistry, Faculty of Chemistry, University of Bucharest, Panduri Av. 90-92, District 5, 050663 Bucharest, Romania; mihaela.buleandra@g.unibuc.ro (M.B.); codreanu.silvia233@gmail.com (S.N.C.); croitoru.lorelei@gmail.com (L.C.); lauraiordache24@gmail.com (L.A.I.); 2 Department of Surgery, Faculty of Medicine, “Lucian Blaga” University Sibiu, Lucian Blaga Street 25, 550169 Sibiu, Romania; hassan.noor@ulbsibiu.ro; 3 European Hospital Medlife-Polisano, Strada Izvorului 1A, 550169 Sibiu, Romania

**Keywords:** ferulic acid, voltammetry, pencil graphite electrode, disposable electrode, cosmetic powder

## Abstract

Ferulic acid (FA), a monohydroxycinnamic acid, is an antioxidant with multiple beneficial effects on human health, presenting also importance in the food and cosmetics industry. Its electrochemical behavior was investigated at the disposable and cost-effective pencil graphite electrode (PGE). Cyclic voltammetry emphasized its pH-dependent, diffusion-controlled oxidation. Using the optimized conditions (HB type PGE, Britton Robinson buffer pH 4.56) differential pulse and square-wave voltammetric techniques were applied for its quantitative determination in the range 4.00 × 10^−7^–1.00 × 10^−3^ mol/L FA. The developed methods were employed for the rapid and simple assessment of the FA content from a commercially available powder designed for cosmetic use.

## 1. Introduction

Ferulic acid (FA, 3-(4-hydroxy-3-methoxyphenyl)prop-2-enoic acid or 4-hydroxy-3-methoxy cinnamic acid) was named after the plant (*Ferula foetida*) from which it was extracted for the first time in 1866 [1]. FA is a phenolic compound, more specifically a derivative of the cinnamic acid, and contains in its structure a benzene ring with two functional groups (methoxy and hydroxyl), as well as a carboxylic moiety [2] (Figure S1). These structural characteristics are probably responsible for most of FA’s properties. Thus, FA is a powerful natural antioxidant, having a strong scavenger effect for various free radicals (hydroxyl, superoxide, hydrogen peroxide and nitrogen dioxide) [3].

In the various studies published over the years, it has been proven that FA plays a crucial role in the human body and has countless beneficial effects: anti-inflammatory, antimicrobial, antiviral, antifungal, vasodilatory, antithrombotic, antiarrhythmic, hepatoprotective, anticarcinogenic, antiallergic, analgesic, neuroprotective, cholesterol-lowering, potential antidepressant, reducing the risk of chronic diseases as diabetes, and skin-protective (enhances wound healing) [3,4,5,6,7,8,9]. FA helps increase sperm viability, is used as food additive, as a component in nutraceuticals and is a constituent in cosmetics and dermatological products [4,9,10,11]. On the other hand, it is worth mentioning that FA is a precursor for the production of vanillin, a flavor compound mainly used in food industry [10]. This is particularly important considering that recently more and more emphasis is placed on the consumption of natural products.

FA is an ubiquitous compound in plant cell walls, constituent of lignocellulose, occurring as a result of the phenylalanine and tyrosine metabolism by the shikimic acid pathway, being found free, dimerized or esterified [1,3,4,10]. FA can be extracted from manifold plants (*Poaceae*, *Solanaceae*, *Chenopodiaceae*, *Amaranthaceae*, *Ranunculaceae*, *Gramineae*, *Ligusticum chuanxiong* Hort., *Ferula asafoetida* L., *Ziziphus jujuba* Mill. var. spinosa, *Angelica sinensis* (Oliv.) Diels, *Cimicifuga foetida* L., parsley, bamboo, and fodder plants), vegetables (spinach, artichoke, rhubarb, sugar beet, carrot, and peanut), fruits (grape, banana, orange, grapefruit, berries, pineapple, pomegranate, pistachio shells, tomato, and nuts), flowers, cereals (rice, wheat, barley, oats, rye, sweet corn, rice bran, and whole grains), beverages (coffee, and beer) [1,4,6,9,10,12]. Moreover, FA is present in some agro-industrial secondary products, for example, corn fiber used for animal feed [11].

Details regarding the identification, biological sources and metabolism of FA, but also challenges associated with FA biopharmaceutical delivery, along with its numerous applications, were addressed in a recent review [4], thereby proving the importance of this compound, as well as the continuous interest shown towards it. Consequently, FA is a significant bioactive compound, representing a trace element essential in life, its identification, characterization and quantification becoming indispensable [13].

Usually, FA is determined employing analytical procedures based on spectrometric (within mixtures) and chromatographic (after separation) methods [2,12]. These have some established strong points, this statement being supported by the fact that the European Pharmacopoeia [14] mentions FA quite often, for instance, as reference or internal standard for the liquid chromatographic assay of herbal species. But, at the same time, these methods also have drawbacks that cannot be overlooked (they are expensive, time consuming, are not suitable for field analysis, involve sample preparation steps, and use toxic solvents).

Taking into account the previously mentioned aspects, the necessity of further developing fast, simple and reliable analytical methods for FA determination from different real samples is self-evident. Within this context, given their multiple advantages (cost-effective, simple, rapid, versatile, characterized by convenient analytical performance parameters, suitable for routine analysis), electrochemical techniques such as cyclic voltammetry (CV), differential pulse voltammetry (DPV) and square-wave voltammetry (SWV) represent a viable option to rely on in order to elaborate useful tools applied for achieving the above-specified objectives. On this line, CV and DPV were applied to study the electrochemical redox mechanism of four polyphenols (gallic acid, caffeic acid, vanillic acid and FA) [15]. The analytes’ redox potentials were used to assess their antioxidant properties, which are useful in pharmaceutical applications.

Next, to go one step further in this endeavor, a well-known thing must be highlighted, namely the importance of the electrode material when a certain analyte is examined. For example, electrochemical sensors based on nano-materials were extensively applied for natural antioxidant determinations in food and biological samples, including FA. A recent review addressed this topic in detail and exhibited the performances, the limitations and the perspectives of this technology [13]. Also, in this regard, disposable pencil graphite electrodes (PGEs) are easily available and constitute useful working electrodes employed for diverse organic and inorganic species determination from various matrices due to their avails: they are cheap, eco-friendly and chemically inert, have uniform surface and mechanical resistance, possess amendable electroactive surface area, present low background currents, and can be used over a wide potential range [16,17,18].

Bearing in mind the benefits of both electrochemical techniques and PGEs, they were combined to study the electrochemical behavior of FA and its quantitative determination. Thus, this paper presents for the first time the results of FA voltammetric analysis at an unmodified, disposable sensor and discusses it in comparison to similar reports previously published in the literature. To the best of our knowledge, the composite graphite material used in the PGE was not exploited till now for FA electroanalysis, despite the fact that this electrode has a very large applicability in voltammetry, including the polyphenolics analysis [19], due to its positive above-mentioned electrochemical and economic advantages [16,17,18]. Moreover, often using unmodified PGE, analytical performances similar to those reported for modified electrodes are obtained, but with a smaller number of steps and a lower consumption of reagents.

## 2. Materials and Methods

FA (≥99%), caffeic acid (≥98%, HPLC), gallic acid (ACS reagent, ≥98.0%), naringin (≥98%, HPLC), ethanol (absolute for analysis EMSURE^®^ACS, ISO, Reag. Ph Eur), boric acid (ACS reagent, ≥99.5%), phosphoric acid (ACS reagent, ≥85 wt. % in H_2_O), hydrochloric acid (ACS reagent, 37%), acetic acid (glacial, ACS reagent, ≥99.7%), NaOH (anhydrous pellets, reagent grade, ≥98%), sodium acetate (anhydrous for analysis EMSURE^®^ACS, ISO, Reag. Ph Eur), di-potassium hydrogen phosphate and potassium dihydrogen phosphate (anhydrous for analysis EMSURE) were Sigma-Aldrich products purchased from Merck (Romania). Double-distilled water was employed for vessel rinsing and solution preparation if not stated otherwise. The 2.00 × 10^−2^ mol/L FA stock solution was freshly prepared daily by dissolving in ethanol the corresponding amount weighed exactly on an analytical balance with four decimal places. The working solutions were obtained by appropriate dilutions with supporting electrolyte from the stock solution, which, when not in use, was kept in the refrigerator. Britton–Robinson buffer (BRB) solutions with various pH values, used as supporting electrolyte, were prepared by adding 0.2 mol/L NaOH solution to the acid mixture (0.04 mol/L H_3_BO_3_, 0.04 mol/L H_3_PO_4_ and 0.04 mol/L CH_3_COOH) until the desired pH was reached. Other employed supporting electrolytes were 0.1 mol/L HCl, acetate buffer solution (ABS) pH 4.00 and phosphate-buffered solution (PBS) pH 7.00.

Powder for cosmetic preparations with a content of 98% FA extracted from the root of the *Ferula assafoetida* L. plant, supplied by Elemental SRL, Oradea, Romania, was purchased from a local drug store and used to test the analytical applicability of the developed voltammetric method. The FA content of the commercially available powder was assessed applying the standard addition method. The amount of powder necessary for the preparation of an FA solution with the theoretical concentration of 3.35 ×10^−3^ mol/L was accurately weighed and dissolved with ethanol in a 10 mL volumetric flask. An aliquot of 0.06 mL from this solution was diluted with BRB pH 4.56 solution to obtain 10 mL of working solution which was analyzed by DPV at PGE. Differential pulse voltammograms were recorded for the 10 mL sample solution before and after each of the four additions of 0.05 mL of 4.00 × 10^−3^ mol/L FA intermediary stock solution and the corresponding obtained peak currents were used to determine the FA concentration in the analyzed sample.

The 0.5 mm Rotring pencil graphite leads that acted as an active surface of the working electrode were purchased at once from a local bookstore.

The pH of the solutions was measured using a combined pH-sensitive glass electrode connected to a pH/mV-meter Consort P901 Scientific Instrument (Belgium).

The voltammetric measurements were made at an Autolab PGSTAT 12 with a GPES 4.9 software, as well as a Voltalab PST 050 Radiometer with a VoltaMaster 4.0 software. For the voltammetric recordings, the electrochemical cell contained 10.00 mL solution to be analyzed, in which the Ag/AgCl/KCl (3.00 mol/L) reference electrode, the platinum wire counter electrode and the PGE working electrode (if not stated otherwise) were immersed. The solid electrodes consisting of glassy carbon (GCE) and platinum (Pt) with a diameter of 0.30 cm and the corresponding geometrical surface area (A_g_) of 0.0707 cm^2^ were employed for comparison. The PGE (Figure S2) was prepared as described in a previous paper [19] and every time, 1.00 cm of the graphite lead was introduced in the solution so that the A_g_ was always constant (0.1589 cm^2^), thus ensuring the reproducibility of the measurements.

Cyclic voltammetry (CV) was used to investigate the voltammetric behavior of FA while, due to their higher sensitivity, differential pulse voltammetry (DPV) and square-wave voltammetry (SWV) were exploited for FA quantification.

## 3. Results and Discussion

Like any analytical study, the present one involved the optimization of various parameters for the FA determination and the investigation of its voltammetric behavior at the disposable PGE, having as the final goal the development of a sensitive method for its simple and rapid quantification in real samples.

### 3.1. Optimization of the Working Conditions for FA Voltammetric Analysis

#### 3.1.1. Selection of the Working Electrode Material

**Figure 1:**
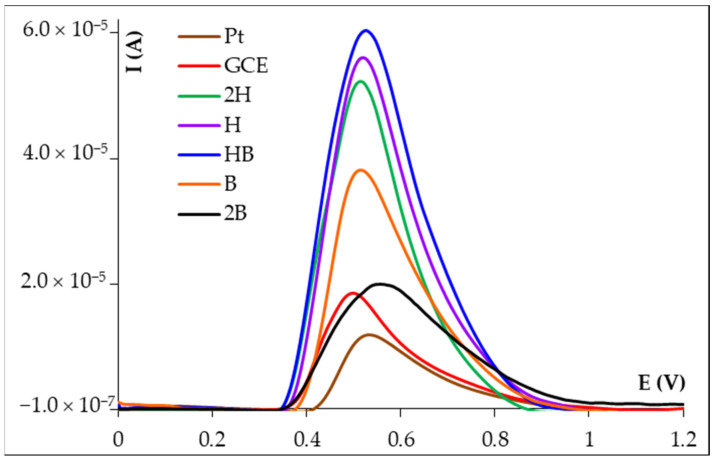
Differential pulse voltammograms recorded at different electrodes for 1.00 × 10^−3^ mol/L FA in ABS pH 4.00.

The choice of PGE as a working electrode was based on the fact that graphite pencil leads have good electrochemical characteristics and by simply changing the pencil lead, a new electroactive surface can be ensured and thus the time-consuming cleaning step of the electrode surface is eliminated. The main components of graphite pencil leads are graphite and resin or high polymer. Depending on the ratio between these two components, there are several types of leads which present different hardness. The softer ones, labeled with B (from blackness) have a higher graphite content, while the harder ones, marked with H (from hardness) contain more polymer or resin [17]. The FA voltammetric response on the classical solid working electrodes (GCE and Pt) as well as on various types of disposable PGEs (Figure 1) emphasized that the highest DPV signal corresponding to the FA oxidation was recorded at the HB type PGE (HB_PGE). The sensitivity (S, expressed in A × L/mol × cm^2^) of the electrodes, an analyte-concentration- and A_g_-electrode-independent parameter, decreased in the order HB_PGE (0.3857) > H_PGE (0.3564) > 2H_PGE (0.3340) > GCE (0.2637) > B_PGE (0.2438) > Pt (0.1742) > 2B_PGE (0.1248).

The selectivity and sensitivity of the voltammetric determinations are strongly influenced by the nature of the electroactive surface, which, most often, is modified in order to improve these characteristics. A simple procedure performed to change the electrode surface is the electrochemical pretreatment, which consists of applying either very negative or very positive potentials or scanning the potential between certain potential values. During this process, the surface is cleaned and oxygen-containing functional groups are created on it. However, the improvements brought by this treatment step to the voltammetric behavior of the analyte depend on both the analyzed species and the chemical and electrochemical applied conditions. FA oxidation signals recorded at HB_PGE pretreated electrochemically either potentiostatic (E constant) or potentiodynamic (CV) using three different supporting electrolytes were compared with that obtained at bare, untreated HB_PGE. As it can be observed from Table 1, the electrochemical pretreatment of the working electrode did not result in an improvement of FA anodic peak current. Therefore, for all following voltammetric investigations, an untreated HB_PGE was employed as the working electrode.

#### 3.1.2. The Stability of the FA Solution

Since antioxidants are chemical species susceptible to being easily oxidized by atmospheric oxygen, both the stability of the FA stock and working solutions were studied. Thus, the variation of FA maximum anodic peak current was monitored during a week by recording the DPV curves for working solutions, freshly prepared at different time intervals, from the same stock solution kept in the refrigerator, when it was not in use. The results showed that the FA oxidation peak intensity slightly increased during the first three days being followed by a decrease (Figure S3). Although the peak current enhancement was only 2.57% one day after the preparation of the FA stock solution, for all subsequent measurements, a stock solution prepared on the day of the determinations was used. Voltammetry has the great advantage that it allows several measurements to be performed at the same solution without changing the concentration of the analyte, and this can only be achieved if the solution in the voltammetric cell is stable under the working conditions (usually ambient). In this context, the DPV anodic currents of FA recorded for 100 min on the same solution were almost constant (Figure S4), indicating that the working solution is stable enough to allow a large number of recordings (at least 50) to be made.

#### 3.1.3. Influence of the Solution pH

Most often, the electrode processes of organic compounds involve, besides the electrons, protons. Therefore, the voltammetric behavior of the analyte depends on the solution pH and consequently the investigation of this chemical parameter is an important issue in the development of electroanalytical methods aimed for the quantitative determination of the corresponding species. The influence of the supporting electrolyte pH on FA voltammetric behavior at HB_PGE was investigated in the pH range 1.81 to 11.92 using the universal BRB and applying CV, DPV and SWV.

**Figure 2:**
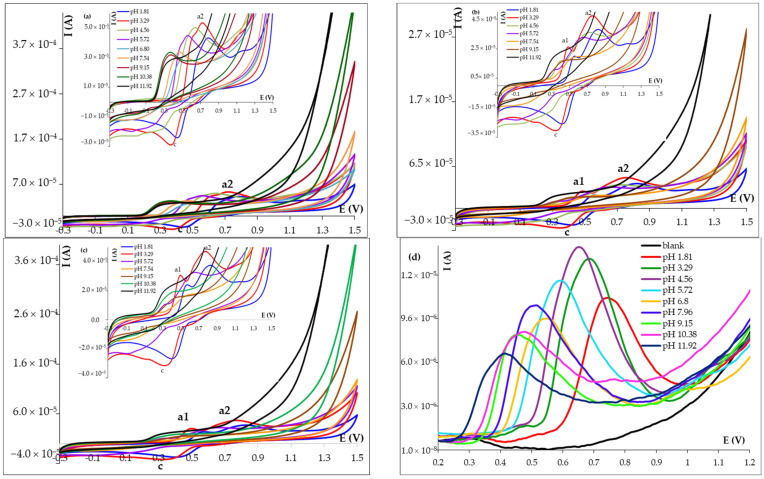
Cyclic voltammograms (**a**) first scan; (**b**) second scan (selection); and (**c**) third scan (selection) of 5.00 × 10^−4^ mol/L FA solutions (scan rate v 0.100 V/s); and (**d**) square-wave voltammograms of 1.00 × 10^−4^ mol/L FA in BRB solutions with different pH values recorded at HB_PGE.

Cyclic voltammetric recordings emphasized that in the first direct scan, one anodic wave (a2) appeared (Figure 2a), while starting with the second forward scan, an additional smaller oxidation peak (a1) can be observed at less positive potentials (Figure 2b,c), up to a pH of about 8.00. Regardless of the scan number, a cathodic signal (c) appeared during the reversed potential sweep. This behavior was similar to that reported by Malagutti et al. [20] according to which the main oxidation signal (a2) corresponded to a two-step oxidation of the guaiacol moiety (similar to GCE, at HB_PGE, the two steps did not give distinct signals, only a small pre-wave can be observed at pH values below approximately 6). The cathodic signal was attributed to the reduction of the previously formed o-quinone, whose oxidation generated in the subsequent scans the anodic signal a1, corresponding to the quasi-reversible quinone–hydroquinone couple (the peaks a1 and c had close potentials, but the current of the cathodic peak was much higher than that of the oxidation signal).

The DPV and SWV recordings (Figure 2d) showed only one oxidation peak during the entire investigated pH range. However, all three applied electrochemical techniques demonstrated that the FA voltammetric peaks shifted towards less positive potentials with the increase of the solution pH, indicating the participation of protons in the corresponding electrode reactions. The variations of the peak potential (E_p_) of a2 signal vs. the solution pH were linear in the pH range 1.81–9.15, while for a1 and c, linear E_p_ = f(pH) dependencies were obtained in the range 1.81–7.96. The corresponding regression equations for each of the FA voltammetric signals showed slopes near to the theoretical value of 0.0592 V/pH from the Nernst equation, suggesting that in each considered electrode reaction, the number of exchanged electrons was equal to that of protons (Table 2).

The highest current intensity of the FA main voltammetric signal (a2) was obtained in the pH range 3.29–4.56, while the more sensitive voltammetric techniques, DPV and SWV (Figure 2d), pointed out that pH 4.56 was the optimum value for FA quantitative determination at HB_PGE and therefore all subsequent measurements were performed in this medium.

### 3.2. Investigation of FA Voltammetric Behavior at HB_PGE

Cyclic voltammetry is the frequently chosen technique for studying the voltammetric behavior of an analyte in certain conditions (in this case, at HB_PGE in BRB pH 4.56) because it provides information about the type and the reversibility of the redox process in which the species of interest participates, as well as on the number of involved electrons and the rate-limiting step.

**Figure 3:**
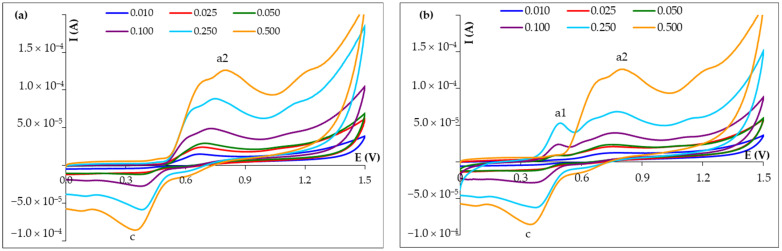
Cyclic voltammograms recorded at HB_PGE for 5.00 × 10^−4^ mol/L FA in BRB pH 4.56 solution at different scan rates: (**a**) first scan; (**b**) second scan.

In order to clarify the voltammetric behavior of FA at HB_PGE, the first two scans were recorded applying different scan rates (v). The cyclic voltammograms corresponding to the first potential scan (Figure 3a) presented two peaks, an oxidation one (a2) at potentials around 0.600–0.700 V and a cathodic signal (c) at 0.350–0.400 V. The positions of these peaks indicated that they did not belong to the same redox couple, so that FA was irreversibly oxidized in the process generating the peak a2. In the second potential scan (Figure 3b), a new anodic peak (a1) appeared at about 0.400–0.450 V, which can be considered to be the counter-partner of the reduction signal (c). These correspond to a redox couple [20] that is quasi-reversible, due to the fact that the peak separation was more than 0.059/n V (n represents the number of electrons involved in the process) and the ratio of the peak currents (I_pa1_/I_pc_) was sub-unitary, the reduction reaction being faster.

From Figure 3, it can be seen that the heights of FA voltammetric signals increased and their potentials shifted (towards more positive values for the anodic peaks and towards less positive potentials for the cathodic one), this last observation being characteristic for quasi-reversible and irreversible electrode processes. The nature and thus the limiting step of the electrode process corresponding to each peak were derived from the various dependencies of the peak height on the scan rate (Table 3). Hence, the FA main oxidation signal a2 was due to a diffusion-controlled process, while the electrode reactions corresponding to the redox couple generating the a1-c peaks pair were controlled by the analyte adsorption at the electrode surface.

At high scan rates, FA oxidation signal a2 seems to represent the sum of two unresolved signals (Figure 3). According to the literature data [20,21], this behavior would be due to a two-step process of one electron each, during which radical species are formed and can further be involved in electropolymerization reactions [22].

**Figure 4:**
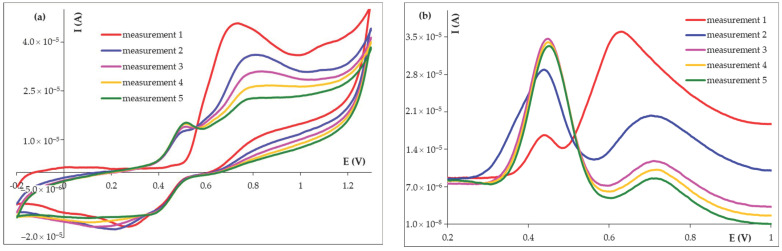
Successive cyclic voltammograms (**a**) and square-wave voltammograms (**b**) recorded at the same HB_PGE for 5.00 × 10^−4^ mol/L FA in BRB pH 4.56 solution.

Repetitive voltammetric measurements performed at the same graphite pencil lead (Figure 4) showed that the anodic peak a2 was diminished with the increase of scan number (Figure 4a). For example, in the second and third SWV recordings, the peak decreased by 70% and 10%, respectively. Starting with the second scan, the anodic signal a1 appeared at less positive potentials and its intensity was enhanced by successive potential sweeps, this fact being further amplified in the square-wave voltammograms (Figure 4b), where the peak intensity was almost doubled in the second scan, while it increased with about 20% during the third scan. This observation, combined with the surface-confined character of the processes corresponding to the a1-c peaks pair, as well as the previously reported results [22], led to the conclusion that during the voltammetric measurements, FA electropolymerization took place. As a result, most probably a weak conductive polymeric film (due to the reduced increase of the a1 and c peaks intensities in the cyclic voltammograms) arose at the electrode surface, hindering the electron transfer between FA and the PGE surface, a fact reflected in the lowered intensity of peak a2. Thus, a new graphite lead must be used for each voltammetric measurement. Fortunately, due to the fact that PGE is disposable and cheap, this is not a disadvantage.

### 3.3. Voltammetric Quantitative Analysis of FA at HB_PGE

#### 3.3.1. Linear Range, Limits of Detection and Quantification

**Figure 5:**
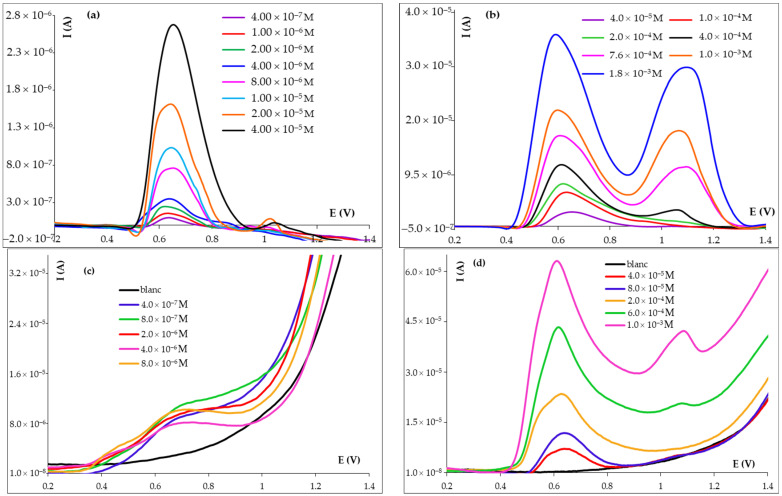
Differential pulse (**a**,**b**) and square-wave (**c**,**d**) voltammograms recorded at HB_PGE for different concentrations of FA in BRB pH 4.56 solutions.

The influence of the FA concentration on the intensity of its main oxidation peak a2 was investigated in the range 1.00 × 10^−7^–2.00 × 10^−3^ mol/L FA using the more sensitive voltammetric techniques, namely DPV and SWV (Figure 5). It can be observed that at higher FA concentrations, a third anodic signal appeared at more positive potentials (≥~1.00 V), but its I_pa_ = f(c) was not investigated. The height of the anodic signal a2 increased with the enhancement of the FA concentration from 4.00 ×10^−7^ to ~1.00 × 10^−3^ mol/L, but the linear dependence between the peak current (I_pa2_) and the analyte concentration was divided into two segments with different slopes. Thus, the calibration graph presented two linear ranges (Table 4) described by the following regression equations (Figure S5): I_pa2_ (A) = 0.0637 C (mol/L) + 2.00 × 10^−7^ (R^2^ = 0.9993) and I_pa2_ (A) = 0.0148 C (mol/L) + 5.00 × 10^−6^ (R^2^ = 0.9987) for DPV and I_pa2_ (A) = 0.1144 C (mol/L) + 2.00 × 10^−6^ (R^2^ = 0.9997) and I_pa2_ (A) = 0.0406 C (mol/L) + 1.00 × 10^−5^ (R^2^ = 0.9993) for SWV. The limits of detection (LOD) (Table 4) and quantification (LOQ) were calculated as 3.3 σ/b and 10 σ/b, respectively, where σ represents the standard deviation of the intercept of the regression equation of the lower linear range and b was the slope (0.0637 A×L/mol for DPV and 0.1144 A × L/mol for SWV) of the calibration curve corresponding to that concentration range. The obtained LOQs were 9.33 × 10^−7^ and 5.52 × 10^−7^ mol/L FA for DPV and SWV, respectively. According to the values of the slopes of the calibration graphs, SWV was more sensitive than DPV, but the performance characteristics of the two techniques are somewhat similar. Compared to other methods developed for FA voltammetric quantification, those presented in this paper, which use bare carbon-based electrodes, have linear ranges (of almost three orders of magnitude) and LODs (at 10^−7^ mol/L level) similar or better than half of those reported in the literature till now (Table 4), all of them using modified working electrodes.

#### 3.3.2. Reproducibility of the Electrode Response

The reproducibility of the HB_PGE voltammetric response expressed as a percentage relative standard deviation (RSD%) and calculated as a standard deviation × 100/average of ten measurements was estimated by both DPV and SWV at three concentration levels situated within the linear range of the methods, each recording being carried out on a new HB_PGE. The RSD% values (Table 5) obtained for each tested concentration fell within the corresponding accepted limits [55], thus demonstrating the good precision of the DPV and SWV methods developed for FA quantification at HB_PGE.

RSD% values from other reported methods can be significantly compared with those obtained in our work only if they were given for the same concentration level. However, a search of the RSD% values reported in the papers published for FA voltammetric analysis revealed the fact that the RSD% values obtained in our study are similar to many of the previously published ones [26,29,31,37,41,43,46], while there are also some with lower RSD% values [20,34,35,44].

### 3.4. Investigation of Possible Interferents on FA Voltammetric Analysis at HB_PGE

The effect of some polyphenolic antioxidants on FA voltammetric analysis at HB_PGE was investigated in BRB pH 4.56 solution. From the huge number of phenolic phytochemicals, which can coexist with FA in various matrices, gallic acid, a hydroxybenzoic acid most commonly used as reference to express the total phenolic content of samples, the structurally related hydroxycinnamic acid, caffeic acid and the glycosylated bioflavonoid naringin (Figure S6) were tested as possible interferents in FA determination by DPV at HB_PGE at molar concentration ratios FA/interferent of 1:1 and 1:10. All examined polyphenols presented oxidation peaks at potentials quite different from that of FA (Figure S7), namely caffeic acid and gallic acid at about 0.300 V and naringin at ~0.890 V and ~1.150 V. Thus, in the given working conditions the investigated antioxidants did not significantly influence the FA oxidation peak current due to the fact that the separation of their peak potentials was about 0.300 V even if the FA anodic peak potential (~0.635 V) was shifted with about 0.03–0.04 V in the negative direction in the presence of caffeic acid and gallic acid and with 0.02 V towards more positive values when it was mixed with naringin. Moreover, it must be specified that the FA signal did not vary more than ±5% in the presence of the tested interferents.

### 3.5. Analytical Application of DPV and SWV at HB_PGE Method for FA Voltammetric Analysis

**Figure 6:**
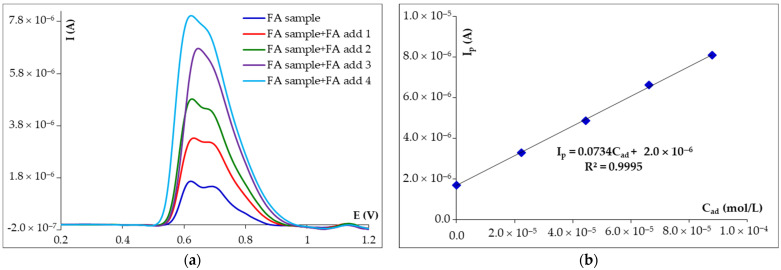
(**a**) Differential pulse voltammograms recorded at HB_PGE for 10 mL FA powder sample in BRB pH 4.56 solution before and after each addition of 0.05 mL of 4.00 × 10^−3^ M FA solution, and (**b**) the corresponding calibration graph.

The analytical applicability of the developed voltammetric methods for rapid FA voltammetric analysis was tested using, as real sample, a commercially available powder for cosmetic preparations with a content of 98% FA extracted from the root of the *Ferula assafoetida* L. The sample working solution was prepared in BRB pH 4.56 solution as previously described in the Experimental section. Differential pulse (Figure 6) and square-wave voltammograms (not shown) recorded for 10 mL powder sample solution before and after each of the four additions of FA solution with exactly known concentration presented only the signal characteristic for the analyte, indicating that there are no interfering species. In both cases, this signal increased linearly with the addition of FA, the corresponding currents being used to determine the FA concentration based on the standard addition method. Thus, the FA recoveries (%) from tested samples were 101.00 ± 2.40% and 99.85 ± 2.76% by DPV and SWV, respectively. The results suggested that the proposed voltammetric methods using HB_PGE are suitable for FA quantification in real samples.

## 4. Conclusions

The present paper describes for the first time the electroanalysis of FA at the unmodified, single-use and cost-effective PGE whose electroactive surface area is a composite graphite-based material. The results were discussed in correlation with those previously reported in the literature and FA voltammetric behavior was similar with that already described for other working electrodes. The use of the HB_PGE in FA voltammetric quantification by both DPV and SWV presented wide linear concentration ranges of more than three orders of magnitude, which are comparable [20,37,39,42,43,45,47,51,53] or even better than almost all (with four exceptions) [15,23,24,25,26,27,28,33,34,35,36,38,40,41,44,46,48,49,50,52,54] voltammetric methods, using modified electrodes reported to date for the FA quantitative determination (Table 4). The LOD values of the voltammetric methods employing the bare PGE for FA detection were similar [25,26,40,41,51,53,54] or even better [27,33,36,38,47,52] than about 1/3 of the 35 papers (Table 4) published strictly on FA voltammetric analysis on modified sensors. Moreover, the selectivity of the bare PGE towards other commonly encountered natural polyphenols was good and last, but not least, the analysis was simple, rapid and involved few, non-toxic reagents so that the methods can be considered to be eco- and user-friendly. The analytical applicability of the HB_PGE was confirmed by the good recoveries generated for FA quantification in a commercially available powder for cosmetic use so that it proved to be a valuable tool for the quality control of various products with not very complicated matrices and could be employed in their routine analysis.

## Figures and Tables

**Table 1:** Anodic peak currents (I_pa_) obtained by DPV for 2.00 × 10^−4^ mol/L FA in ABS pH 4.00 using untreated and electrochemically pretreated HB_PGEs.

	Electrochemical Pretreatment	E Constant (2.00 V; 60 s)	CV (−2.00 to 2.00 V; Can Rate 0.500 V/s; 10 Cycles)	None
Medium		I_pa_ (A)		
0.1 mol/L HCl		1.82 × 10^−5^	1.74 × 10^−5^	1.87 × 10^−5^
ABS pH 4.00		1.57 × 10^−5^	1.85 × 10^−5^	
PBS pH 7.00		1.67 × 10^−5^	1.47 × 10^−5^	

**Table 2:** The regression equations and the determination coefficients (R^2^) for the E_p_ = f(pH) dependencies of each voltammetric signal of FA observed at HB_PGE.

Technique	pH Range	a1 Signal	a2 Signal	c Signal
CV scan 1	1.81–9.15		E_pa2_ = −0.0566 × pH + 0.8747 (R^2^ = 0.9998)	E_pc_ = −0.0566 × pH + 0.8747 (R^2^ = 0.9998)/1.81–7.96
CV scan 2	1.81–7.96	E_pa1_ = −0.0569 × pH + 0.6467 (R^2^ = 0.9960)	E_pa2_ = −0.0494 × pH + 0.8998 (R^2^ = 0.9965)	E_pc_ = −0.0566 × pH + 0.8747 (R^2^ = 0.9998)
CV scan 3	1.81–7.96	E_pa1_ = −0.0574 × pH + 0.6575 (R^2^ = 0.9979)	E_pa2_ = −0.0498 × pH + 0.9201 (R^2^ = 0.9924)	E_pc_ = −0.0566 × pH + 0.8747 (R^2^ = 0.9998)
DPV	1.81–9.15		E_pa2_ = −0.0504 × pH + 0.8198	
			(R^2^ = 0.9750)	
SWV	1.81–9.15		E_pa2_ = −0.0505 × pH + 0.8633	
			(R^2^ = 0.9959)	

**Table 3:** Various dependencies of the FA peak currents on the potential scan rate corresponding to the cyclic voltammograms from Figure 3.

Dependence		Regression Equation		
	Peak	a1 (E_pa1_ ~0.450–0.500 V)	a2 (E_pa2_ ~0.700–0.800 V)	c (E_pc_ ~0.350–0.400 V)
Scan 1				
I_p_ = f(v)			Non-linear	I_pc_ = 2.00 × 10^−4^ × v − 9.00 × 10^−9^ (R^2^ = 0.9934)
I_p_ = f(v^1/2^)			I_pa2_ = 1.00 × 10^−4^ × v^1/2^ + 3.00 × 10^−6^ (R^2^ = 0.9964)	Non-linear
log I_p_ = f(log v)			log I_pa2_ = 0.5367 × log v − 3.9449 (R^2^ = 0.9931)	log I_pc_ = 1.0536 × log v − 3.7689 (R^2^ = 0.9947)
Scan 2				
I_p_ = f(v)		I_pa1_ = 1.00 × 10^−4^ × v − 8.00 × 10^−7^ (R^2^ = 0.9933)	Non-linear	I_pc_ = −2.00 × 10^−4^ × v + 7.00 × 10^−7^ (R^2^ = 0.9962)
I_p_ = f(v^1/2^)		Non-linear	I_pa2_ = 5.00 × 10^−5^ × v^1/2^ − 4.00 × 10^−7^ (R^2^ = 0.9974)	Non-linear
log I_p_ = f(log v)		log I_pa1_ = 1.1625 × log v − 3.8592 (R^2^ = 0.9871)	log I_pa2_ = 0.5036 × log v − 4.3285 (R^2^ = 0.9934)	log I_pc_ = 1.1620 × log v − 3.6963 (R^2^ = 0.9964)

**Table 4:** The performance characteristics of voltammetric methods reported in the literature for FA determination.

Technique	Electrode	Linear Range (mol/L)	Limit of Detection (mol/L)	Sample	Ref.
CV/LSV	GCE	4.00 × 10^−4^–1.00 × 10^−3^	-	Orange juice	[23]
Amp	CDE	2.57 × 10^−7^–2.57 × 10^−5^	5.15 × 10^−8^	Nao Xue Shuan Tablets	[24]
CV	MWCNTs-GCE	1.00 × 10^−5^–5.00 × 10^−3^	1.00 × 10^−7^	Xiao Yao Pill	[25]
DPV	MWCNTs-GCE	5.30 × 10^−6^–5.30 × 10^−4^	3.20 × 10^−7^	Coffee	[26]
DPV	MWCNTs-GCE	2.00 × 10^−6^–1.00 × 10^−5^	1.00 × 10^−6^	Tablets	[27]
DPV	MWCNTs-GCE	1.03 × 10^−8^–6.17 × 10^−7^	-	-	[15]
CV	GO/MWCNT-GCE	2.40 × 10^−7^–3.20 × 10^−5^ 8.80 × 10^−5^–1.23 × 10^−3^	8.00 × 10^−8^	*Pinellia ternate*	[28]
Amp	Ag/MWCNTs-CPE	4.00 × 10^−8^–1.00 × 10^−3^	3.00 × 10^−8^	Spiked urine; wine	[29]
DPV	α-Fe_2_O_3_/MWCNTs-GCE	up to 6.00 × 10^−6^	1.75 × 10^−8^	Tablets	[30]
SWV	MnO_2_-NPs/MWCNTs-GCE	8.20 × 10^−8^–2.20 × 10^−4^	1.00 × 10^−8^	Spiked human serum	[31]
DPV	MgO/SWCNTs-[Bmim][Tf2N]-CPE	9.00 × 10^−9^–4.50 × 10^−4^	3.00 × 10^−9^	Red wine; white rice	[32]
DPV	g-C_3_N_4_/CS-GCE	2.57 × 10^−5^–1.54 × 10^−4^	2.55 × 10^−5^	Food	[33]
DPV	pBCP/f-SWCNTs-GCE	1.00 × 10^−7^–5.00 × 10^−6^ 5.00 × 10^−6^–2.50 × 10^−5^	7.20 × 10^−8^	Vanilla extracts	[34]
DPV	pSY/MWCNTs-GCE	5.00 × 10^−7^–4.00 × 10^−6^	9.80 × 10^−8^	Coffee	[35]
SWV	PPy/MWCNT-GCE	3.32 × 10^−6^–2.59 × 10^−5^	1.17 × 10^−6^	Popcorn	[36]
Amp	GCE PEI/MWCNT/GCE	5.00 × 10^−7^–2.00 × 10^−5^ 1.00 × 10^−7^–1.00 × 10^−4^	2.00 × 10^−7^ 8.00 × 10^−8^	Wines	[37]
CV	EPD-DyPc2 EPD-GdPc2 EPD-LuPc2	6.00 × 10^−5^–1.00 × 10^−4^	2.19 × 10^−6^ 3.18 × 10^−5^ 6.91 × 10^−6^	-	[38]
LSV	pGlu-GCE	2.00 × 10^−7^–3.00 × 10^−4^	7.00 × 10^−8^	Chinese proprietary medicine	[39]
CV	DDAB/Naf-CPE	2.00 × 10^−6^–1.20 × 10^−4^	3.90 × 10^−7^	Pharmaceuticals	[40]
DPV	GN-GCE	5.00 × 10^−7^–5.00 × 10^−5^	2.00 × 10^−7^	Tablets	[41]
DPV	PDDA-G-GCE	8.95 × 10^−8^–5.29 × 10^−5^	4.42 × 10^−8^	*Angelica sinensis;* spiked human urine	[42]
SWV	γ-CoTe_2_-GCE	4.00 × 10^−8^–2.80 × 10^−5^	1.30 × 10^−8^	Cosmetics	[20]
DPV	erGO-GCE	8.49 × 10^−8^–3.89 × 10^−5^	2.06 × 10^−8^	Powder of A. sinensis; spiked urine and plasma	[43]
DPV	rGO-AuNPs/MIP-(a)SPE	1.00 × 10^−8^–1.00 × 10^−6^	3.10 × 10^−9^	Orange peels	[44]
DPV	TiO_2_-NPs/rGO-GCE	1.00 × 10^−7^–3.00 × 10^−4^	1.00 × 10^−8^	Popcorn, corn milk	[45]
SWV	rG-CdO/MOITF-CPE	2.00 × 10^−8^–4.00 × 10^−6^	8.00 × 10^−8^	Wheat flour; corn cider; corn milk	[46]
Amp	G-novolac-CPE	1.00 × 10^−6^–1.00 × 10^−3^	7.50 × 10^−7^	*Cimicifugae rhizoma*	[47]
SWV	rGO/ZrHCF-PIGE	1.14 × 10^−9^–4.20 × 10^−8^	3.80 × 10^−10^	Milk, water	[48]
CA	MoO_3_-CC	3.00 × 10^−5^–3.00 × 10^−4^	2.30 × 10^−9^	Pineapple juice, water	[49]
Amp	La–Ty Sonogel–Carbon biosensor	3.00 × 10^−8^–2.50 × 10^−6^	6.40 × 10^−8^	Beer	[50]
FIA-Amp	L-Cys/SAM-Au	5.00 × 10^−7^–8.00 × 10^−5^ 1.00 × 10^−4^–1.00 × 10^−3^	1.20 × 10^−7^	Sodium ferulate injection	[51]
DPV	Paper-based electrochemical device	1.54 × 10^−5^–7.20 × 10^−4^	5.15 × 10^−6^	Corn cider, corn milk	[52]
SVW	MBIBr/NiO-SWCNTs-CPE	6.00 × 10^−8^–9.00 × 10^−4^	2.00 × 10^−8^	Corn milk, wheat flour, corn cider	[8]
DPV	MnFe_2_O_4_/BMIM-PF_6_-CPE	3.00 × 10^−7^–2.50 × 10^−4^	1.00 × 10^−7^	Blueberry, white rice, mango dried powder	[53]
CV	CNF-SPE	1.00 × 10^−5^–1.00 × 10^−3^	2.33 × 10^−7^	Eye Blend product	[54]
DPV SWV	PGE	4.00 × 10^−7^–1.00 × 10^−4^ 1.00 × 10^−4^–1.82 × 10^−3^ 4.00 × 10^−7^–8.00 × 10^−5^ 1.00 × 10^−4^–1.00 × 10^−3^	3.08 × 10^−7^ 1.82 × 10^−7^		This work

**Table 5:** Results of the reproducibility studies obtained for the voltammetric determination at HB_PGE in BRB pH 4.56 of FA at different concentration levels.

Concentration (mol/L)		RSD%	
	Technique	SWV	DPV
4.00 × 10^−7^		6.36	6.03
2.00 × 10^−6^		4.59	4.42
5.00 × 10^−4^		3.10	3.33

## Data Availability

Data are contained within the article or supplementary material.

## Supplementary Materials

The following supporting information can be downloaded at https://www.mdpi.com/article/10.3390/mi14101951/s1. Figure S1: FA chemical structure; Figure S2: The PGE; Figure S3: The variation of the DPV anodic peak current recorded at HB_PGE for 1.00 × 10^−3^ mol/L FA in ABS pH 4.00 prepared at different days from the same ethanolic 1.00 × 10^−2^ mol/L FA stock solution; Figure S4: The variation of the DPV anodic peak current recorded at different time intervals at HB_PGE for a 5.00 × 10^−4^ mol/L FA in ABS pH 4.00. Figure S5: The Ip = f(C) dependencies obtained for the analysis of FA in BRB pH 4.56 solutions at HB_PGE by (a) DPV and (b) SWV; Figure S6: Chemical structures of the polyphenols tested as possible interferents in FA DPV analysis at HB_PGE (a) gallic acid (GA), (b) caffeic acid (CA) and (c) naringin (NG); Figure S7: Differential pulse voltammograms recorded at HB_PGE for 8.00 × 10^−6^ mol/L FA in BRB pH 4.56 solutions in the presence of (a) gallic acid, (b) caffeic acid and (c) naringin.

## Abbreviations

α-Fe_2_O_3_/MWCNTs	α-ferric oxide/multi-walled carbon nanotubes
Ag/MWCNTs	silver decorated multi-walled carbon nanotubes
Amp	amperometry
CA	chronoamperometry
CC	carbon cloth
CDE	carbon disc electrode
CNF	carbon nanofiber
CPE	carbon paste electrode
CV	cyclic voltammetry
DDAB/Naf	didodecyldimethylammonium bromide/Nafion composite film
DPV	differential pulse voltammetry
EPD-LnPc2 sensors	electrophoretic deposition-lanthanoid bisphthalocyanines voltammetric multisensor
erGO	electrochemically reduced graphene oxide
FIA	flow injection analysis
g-C_3_N_4_/CS	graphite-like carbon nitride/chitosan
GCE	glassy carbon electrode
GN	graphene nanosheet
G-novolac	Graphene–phenolic novolac resin composite
GO/MWCNTs	graphene oxide sheets and multi-walled carbon nanotubes nanocomposite
La–Ty	Laccase–Tyrosinase
L-Cys/SAM	L-cysteine self-assembled monolayers
LSV	linear sweep voltammetry
MBIBr/NiO-SWCNTs	NiO-embedded single-walled carbon nanotube nanocomposite and n-methyl-3-butylimidazolium bromide
MgO/SWCNTs-[Bmim][Tf2N]	magnesium oxide/single-walled carbon nanotubes/1-butyl-3-methylimidazolium bis(trifluoromethylsulfonyl)imide
MnFe_2_O_4_/BMIM-PF_6_	MnFe_2_O_4_ nanoparticle/1-butyl-3-methylimidazolium hexafluorophosphate
MnO_2_-NPs/MWCNTs	MnO_2_ nanoparticles decorated multi-walled carbon nanotubes
MWCNTs	multi-walled carbon nanotubes
pBCP/f-SWCNTs	polybromocresol purple/polyaminobenzene sulfonic acid functionalized single-walled carbon nanotubes
PDDA-G	poly(diallyldimethylammoniumchloride) functionalized graphene
PEI/MWCNT	polyethyleneimine–multi-walled carbon nanotube
PGE	pencil graphite electrode
pGlu	poly(glutamic acid)
PPy/MWCNT	polypyrrole-multiwalled carbon nanotubes
pSY/MWCNTs	poly(Sunset Yellow)/multiwalled carbon nanotubes
rG-CdO/MOITF	reduced graphene decorated CdO nanocomposite/1- methyl-3-octylimidazolium tetrafuoroborate ionic liquid
rGO-AuNPs/MIP-(a)SPE	molecularly imprinted on reduced graphene oxide-gold nanoparticles modified activated screen printed electrode
rGO/ZrHCF-PIGE	reduced graphene oxide/zirconium hexacyanoferrate modified paraffin impregnated graphite electrode
SPE	screen-printed electrode
SWV	square-wave voltammetry
TiO_2_-NPs/rGO	TiO_2_ nanoparticle-decorated, chemically reduced graphene oxide
