# Supplementary material for: Voltammetric Investigation of Ferulic Acid at Disposable Pencil Graphite Electrode
Source: Micromachines (Basel). 2023 Oct 19;14(10):1951. doi: 10.3390/mi14101951 (PMC10609049; doi:10.3390/mi14101951)
Supplement: Supplementary file 1 [file micromachines-14-01951-s001.zip › micromachines-2664007-SI.pdf]

Supplementary information

Voltammetric Investigation of Ferulic Acid at Disposable Pencil Graphite Electrode

Iulia Gabriela David [1,*], Dana Elena Popa [1,*], Mihaela Buleandra [1], Silvia Nicoleta Codreanu [1], Lorelei Croitoru [1], Laura Andreea Iordache [1] and Hassan Noor [2,3]

[1] Department of Analytical Chemistry and Physical Chemistry, Faculty of Chemistry, University of Bucharest, Panduri Av. 90-92, District 5, 050663 Bucharest, Romania; mihaela.buleandra@g.unibuc.ro (M.B.); codreanu.silvia233@gmail.com (S.N.C.); croitoru.lorelei@gmail.com (L.C.); lauraiordache24@gmail.com (L.A.I.)

[2] Department of Surgery, Faculty of Medicine, "Lucian Blaga" University Sibiu, Lucian Blaga Street 25, 550169 Sibiu, Romania; hassan.noor@ulbsibiu.ro

[3] European Hospital Medlife-Polisano, Strada Izvorului 1A, 550169 Sibiu, Romania

* Correspondence: gabrielaiulia.david@g.unibuc.ro (I.G.D.); elena.popa@chimie.unibuc.ro (D.E.P.)

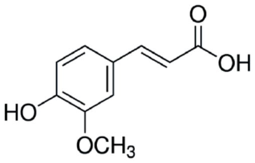

Figure S1. FA chemical structure

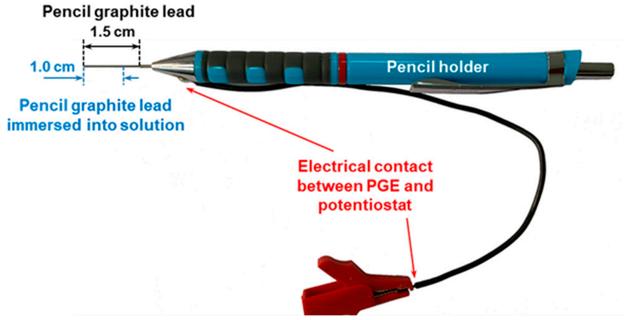

Figure S2. The PGE.

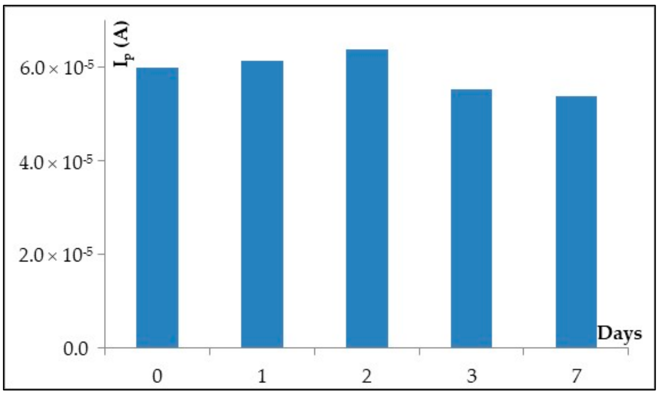

Figure S3. The variation of the DPV anodic peak current recorded at HB_PGE for 1.00 × 10^{-3} mol/L FA in ABS pH 4.00 prepared at different days from the same ethanolic 1.00 × 10^{-2} mol/L FA stock solution.

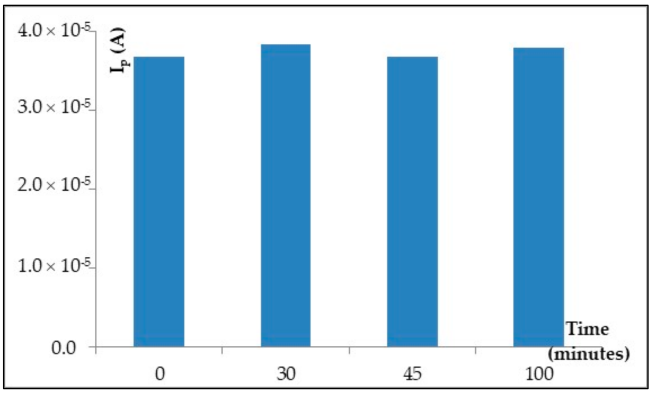

Figure S4. The variation of the DPV anodic peak current recorded at different time intervals at HB_PGE for a 5.00 × 10^{-4} mol/L FA in ABS pH 4.00.

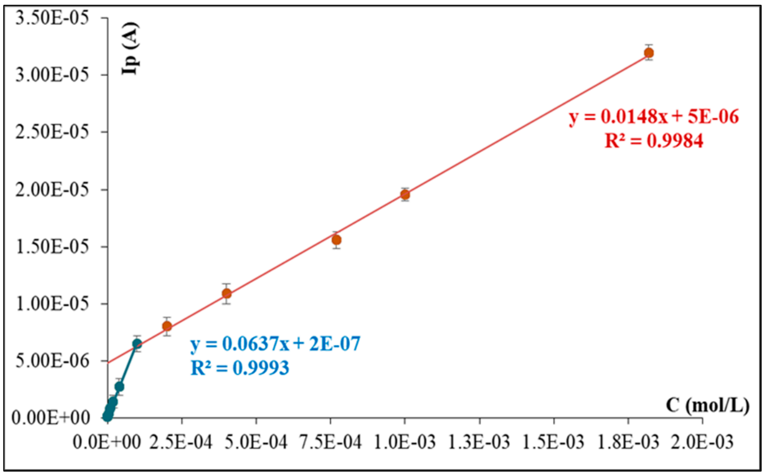

(a)

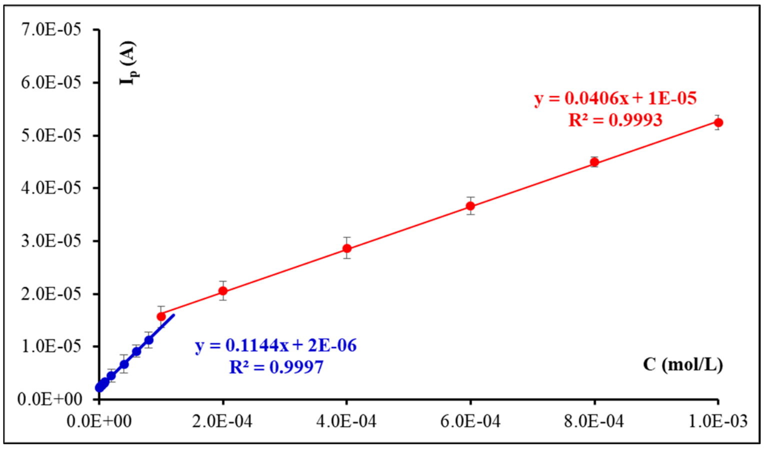

(b)

Figure S5. The Ip=f(C) dependencies obtained for the analysis of FA in BRB pH 4.56 solutions at HB_PGE by (a) DPV and (b) SWV.

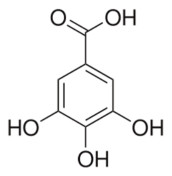

(a)

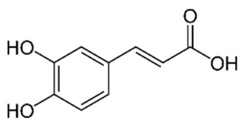

(b)

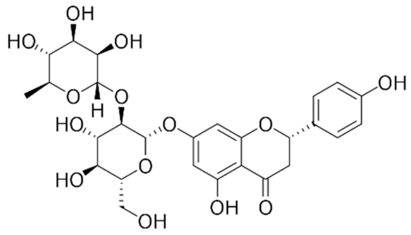

(c)

Figure S6. Chemical structures of the polyphenols tested as possible interferents in FA DPV analysis at HB_PGE (a) gallic acid; (b) caffeic acid and (c) naringin.

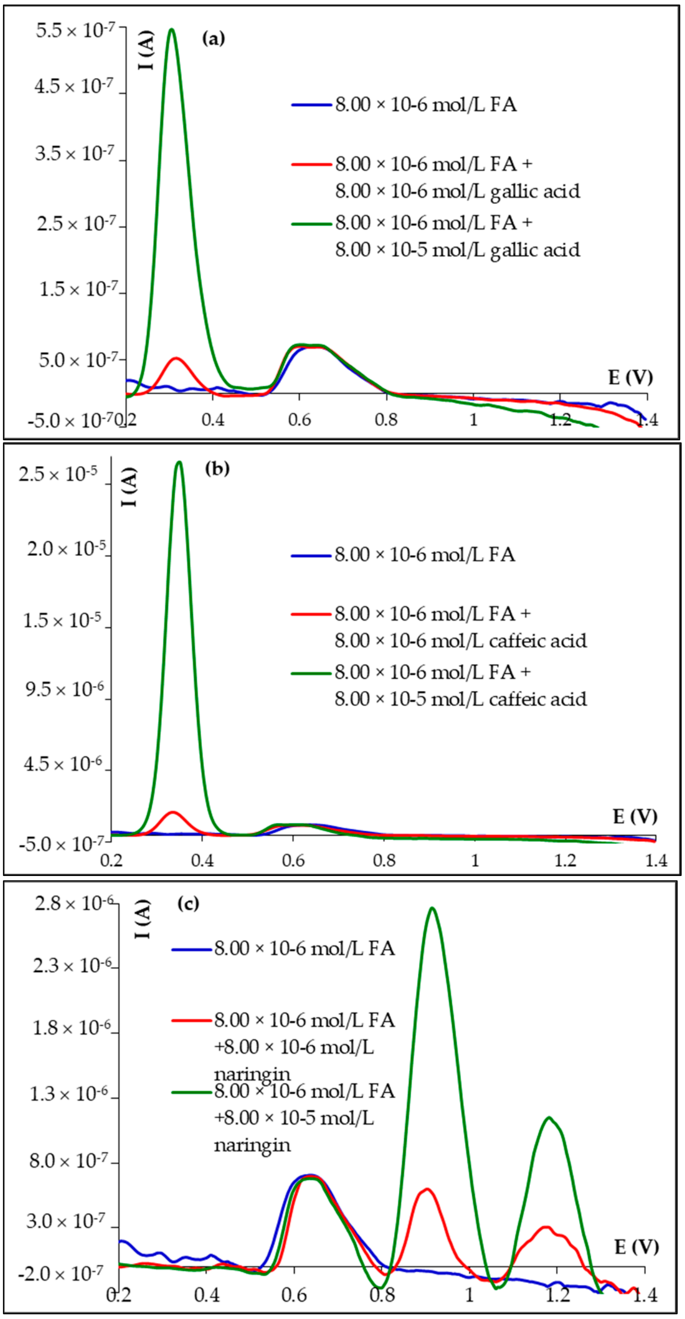

Figure S7. Differential pulse voltammograms recorded at HB_PGE for 8.00×10^{-6} mol/L FA in BRB pH 4.56 solutions in the presence of (a) gallic acid; (b) caffeic acid and (c) naringin.
